# Kinetics of IFNγ-Induced Cytokines and Development of Immune-Related Adverse Events in Patients Receiving PD-(L)1 Inhibitors

**DOI:** 10.3390/cancers16091759

**Published:** 2024-05-01

**Authors:** Leticia Alserawan, Maria Mulet, Geòrgia Anguera, Mariona Riudavets, Carlos Zamora, Rubén Osuna-Gómez, Jorgina Serra-López, Andrés Barba, Ivana Sullivan, Margarita Majem, Silvia Vidal

**Affiliations:** 1 Immunology-Inflammatory Diseases, Biomedical Research Institute Sant Pau (IIB Sant Pau), 08025 Barcelona, Spain; alserawan@clinic.cat (L.A.); mmulet@santpau.cat (M.M.); carlosza86@gmail.com (C.Z.); rosuna@santpau.cat (R.O.-G.); 2 Department of Immunology, Hospital Clínic Barcelona, 08036 Barcelona, Spain; 3 Department of Medical Oncology, Hospital de la Santa Creu i Sant Pau, 08025 Barcelona, Spain; ganguera@santpau.cat (G.A.); mariona.riudavetsmelia@aphp.fr (M.R.); jserral@santpau.cat (J.S.-L.); abarba@santpau.cat (A.B.); isullivan@santpau.cat (I.S.); mmajem@santpau.cat (M.M.); 4 Department of Pneumologie, Hôpital Cochin—APHP Centre, 75014 Paris, France

**Keywords:** interferon, cytokines, immune checkpoint inhibitors, anti-PD-(L)1 inhibitors, immune-related adverse events (irAEs), predictive biomarkers

## Abstract

**Simple Summary:**

Immune checkpoint inhibitors (ICI) have the potential to induce serious and unpredictable immune-related adverse events (irAEs), the underlying mechanisms of which remain incompletely understood. In this study, we investigated the relationship between irAEs and the expression of IFN-inducible chemokines and cytokines in patients with solid tumours treated with PD-(L)1 inhibitors. We analysed plasma levels of various IFN-related cytokines at different time points in patients categorized by irAE development and severity. We found that patients with serious irAEs showed significant increases in CXCL9, CXCL10, CXCL11, IL-18 and IL-10 at the onset of the irAE compared to patients with mild irAEs and those without irAEs. Additionally, IL-18 emerged as a promising predictive biomarker for serious irAE development. In summary, this study provides valuable insights into the immune responses associated with irAEs and proposes potential predictive markers for their severity.

**Abstract:**

Immune checkpoint inhibitors (ICI) have the potential to trigger unpredictable immune-related adverse events (irAEs), which can be severe. The underlying mechanisms of these events are not fully understood. As PD-L1 is upregulated by IFN, the heightened immune activation resulting from PD-1/PD-L1 inhibition may enhance the IFN response, triggering the expression of IFN-inducible genes and contributing to irAE development and its severity. In this study, we investigated the interplay between irAEs and the expression of IFN-inducible chemokines and cytokines in 134 consecutive patients with solid tumours treated with PD-(L)1 inhibitors as monotherapy or in combination with chemotherapy or other immunotherapy agents. We compared the plasma levels of IFN-associated cytokines (CXCL9/10/11, IL-18, IL-10, IL-6 and TGFβ) at various time points (at baseline, at the onset of irAE and previous to irAE onset) in three patient groups categorized by irAE development and severity: patients with serious irAEs, mild irAEs and without irAEs after PD-(L)1 inhibitors. No differences were observed between groups at baseline. However, patients with serious irAEs exhibited significant increases in CXCL9/10/11, IL-18 and IL-10 levels at the onset of the irAE compared to baseline. A network analysis and correlation patterns highlighted a robust relationship among these chemokines and cytokines at serious-irAE onset. Combining all of the analysed proteins in a cluster analysis, we identified a subgroup of patients with a higher incidence of serious irAEs affecting different organs or systems. Finally, an ROC analysis and a decision tree model proposed IL-18 levels ≥ 807 pg/mL and TGFβ levels ≤ 114 pg/mL as predictors for serious irAEs in 90% of cases. In conclusion, our study elucidates the dynamic changes in cytokine profiles associated with serious irAE development during treatment with PD-(L)1 inhibitors. The study’s findings offer valuable insights into the intricate IFN-induced immune responses associated with irAEs and propose potential predictive markers for their severity.

## 1. Introduction

In the latest decade, PD-(L)1 inhibitors have improved the treatment outcome of several types of solid tumours, either in monotherapy or in combination with chemotherapy and/or other immune checkpoint inhibitor (ICI) agents [1]. Their activity is based on the reversion of the immunosuppressive effect caused by the expression and interaction of immune checkpoints as Programmed cell death 1 receptor (PD-1) and its ligands (PD-L1 or PD-L2), after continuous stimulation in the tumour microenvironment.

Despite their efficacy, leukocyte activation produced by these agents can cause adverse events in the form of autoinflammation or autoimmunity, which are commonly named immune-related adverse events (irAEs). irAEs are unpredictable diverse immune-mediated events that can affect any organ or system and can occur at any moment during or after treatment. Up to 80% of patients treated with ICI can experience irAEs and up to 25% of cases can be serious and may require immunosuppressors and eventually treatment discontinuation [2]. In some instances, these toxicities are life-threatening and potentially permanent [3]. For this reason, an early identification of patients who will develop irAEs, especially the most severe forms, is crucial for their prompt management.

Biomarker candidates for irAE diagnosis and prediction are under investigation. Inflammatory cytokines and chemokines play a role in the immune response and can be involved in the development of irAEs when the immune system becomes dysregulated due to ICI therapy [4]. One in particular, interferon gamma (IFNγ) is a key cytokine produced by activated T cells, NK and NKT and plays a critical role in orchestrating antitumor responses [5,6]. IFNγ is involved in the development of autoimmune diseases such as systemic lupus erythematosus, systemic sclerosis and Sjögren’s syndrome, in which affected patients frequently have an increased expression of interferon-stimulated genes (ISGs) in peripheral blood (interferon signature) [7,8,9,10]. Autoinflammatory disorders such as Aicardi-Goutières, CANDLE syndrome and SAVI syndrome also show an elevated IFN signature [11,12]. 

Among the ISGs, there are several chemokines. The IFN-inducible chemokines CXCL9 (also known as monokine induced by gamma interferon or MIG), CXCL10 (interferon γ-induced protein 10 or IP-10) and CXCL11 (interferon-inducible T-cell alpha chemoattractant or I-TAC) play an important role in the proliferation and function of T cells after PD-1 blockade. They promote cell migration and Th1 polarization and activation [13,14,15]. Plasmatic levels of these chemokines after ICI initiation have been correlated with ICI efficacy [16,17]. In addition, some studies have suggested that the occurrence of certain irAEs may be associated with a better response to ICIs [18,19,20]. Patients who experience these irAEs might have a more robust immune response against the tumour. However, this relationship is not consistent across all studies and is still the subject of ongoing research [21,22].

In addition, immune dysregulation may involve the participation of other pro- and anti-inflammatory cytokines. IL-18 is a key pro-inflammatory cytokine of the inflammasome produced by a wide range of innate immune or non-lymphoid cells that contributes to induce Th1-like cell responses. This cytokine has a potent ability to induce IFNγ production and is upregulated in some autoinflammatory diseases [23].

Other cytokines with immunosuppressive activity such as IL-10 or TGFβ have an important role in maintaining tolerance against self and innocuous antigens [24,25]. The dysregulation of these cytokines has been associated with an increased risk of developing autoimmune diseases [26] and may have a role in irAE development.

Our aim was to determine whether the levels of IFN-inducible chemokines throughout the treatment with PD-(L)1 inhibitors are linked with the development of serious irAEs. Initially, we examined the levels of these chemokines in patients who experienced serious irAEs, mild irAEs and who did not experience irAEs during ICI treatment. Subsequently, we explored the relationship between these and other related cytokines at the onset of the irAEs. Lastly, we investigated whether any of these proteins, individually or in combination, could predict serious irAEs and serve as predictive and prognostic biomarkers. 

## 2. Materials and Methods

### 2.1. Patient Inclusion and Clinical Assessment

We prospectively enrolled 134 patients diagnosed with solid tumours and treated with PD-(L)1 inhibitors. This cohort was diagnosed and monitored by the Department of Medical Oncology of Hospital Santa Creu i Sant Pau (Barcelona, Spain) from September 2018. The end of the follow up was September 2020. PD-(L)1 inhibitors were administered either as monotherapy or in combination with chemotherapy or other immunotherapy agents. All patients included received at least one dose of ICI. Treatments were administered according to the standard of care. None of the patients had autoimmune diseases or contraindications for receiving immunotherapy.

Symptoms, physical examination and laboratory data of the patients were evaluated every 3–4 weeks. Thyroid function was assessed at baseline and subsequently every six weeks. IrAEs were defined as adverse events with a potential immunologic basis that required close monitoring and/or potential intervention with immunosuppressive or hormone replacement therapy. The severity of the IrAEs was graded according to the Common Terminology Criteria for Adverse Events (CTCAE) version 5.0 [27]. Patients’ data were collected from electronic medical records. 

**Figure 1:**
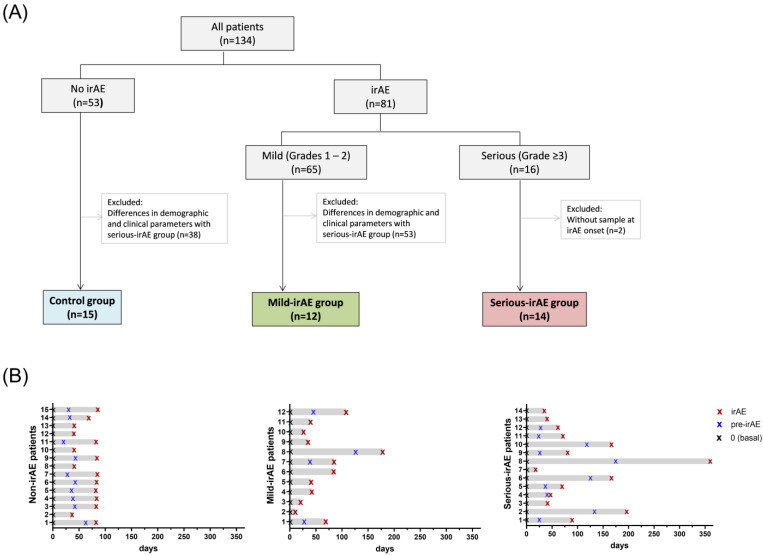
Patient inclusion scheme. (**A**) A total of 134 patients were treated with ICI and prospectively followed-up. We analysed samples from 41 patients: 14 patients with grade ≥ 3 irAEs (serious-irAE), 12 patients with grade 1–2 irAEs (mild-irAE) and 15 patients without any irAE (non-irAE) as the control group, matched in gender, age, tumour and treatment. (**B**) Schematic representation of the collection time points of samples within each group.

For this study, we categorized grade 3 and grade 4 irAEs as serious irAEs, and grade 1 and grade 2 irAEs as mild irAEs. We selected those patients who developed grade 3–4 irAEs with available samples at irAE onset (serious-irAE group, n = 14) and, in accordance with our objective, we also analysed patients who developed grade 1–2 irAEs matched in gender, age, tumour, treatment type and irAE types and frequency with available samples at irAE onset (mild-irAE group, n = 12). Lastly, a control group of patients who had not experienced any irAE and who matched in the same characteristics (gender, age, tumour and treatment type) was also analysed (non-irAE group, n = 15). Figure 1A shows the patient inclusion scheme for this study. For patients experiencing more than one irAE, the most severe type of irAE was considered for this analysis. 

### 2.2. Sample Collection

Serial whole blood samples from each patient were collected in heparinized BD Vacutainer tubes (BD, Franklin Lakes, NJ, USA) at baseline and at 4, 10, 18 and 24 weeks after the initiation of ICI treatment. Additionally, an extra sample was collected when a patient experienced an irAE. All samples were centrifuged at 500 g for 15 min, and plasma was separated and cryopreserved until cytokine determination. In patients who experienced an irAE (mild and serious-irAE groups), pre-treatment samples (baseline), samples at the onset of the irAE development (irAE) and samples collected before the irAE sample (pre-irAE), were analysed (Figure 1B). 

In the serious-irAE group, the median (interquartile range, IQR) days after ICI initiation of irAE samples was 71 (41–167) days and of pre-irAE samples was 39 (24–127) days. Pre-irAE samples were taken 52.5 (22.2–72.7) days before irAE onset. In the mild-irAE group, the median (IQR) days after ICI initiation of irAE samples was 41 (28–84) days and of pre-irAE samples was 41 (29–105). Pre-irAE samples were taken 44 (35–77) days before irAE onset. In the cases where the irAE occurred in the first 4 weeks (4 patients in the serious-irAE group and 8 patients in the mild-irAE group), no pre-irAE samples were available. In the non-irAE group (control group), pre-treatment and two more consecutive samples were selected at equivalent time points to the pre-irAE and irAE samples of the mild and serious-irAE groups. The median (IQR) days after ICI initiation of the first post-treatment sample selected was 36 (29–42) days and the second post-treatment sample was 83 (41–84) days. There were no significant differences between the days after ICI initiation in pre-irAE samples and sample 1, nor between irAE samples and sample 2 of the non-irAE group (*p* = 0.7 and *p* = 0.9, respectively). Due to an early progression observed in 5 patients in the control group, only one post-treatment sample was available. 

### 2.3. Determination of Plasmatic Concentration of Cytokines

Plasma samples were analysed using a commercial customized Milliplex Human magnetic bead panel (Reference code HCYTA-60K, Millipore, Billerica, MA, USA) coupled with the Luminex xMAP platform (Luminex corporation-Diasorin, Saluggia, Italy) for CXCL9 (MIG), CXCL10 (IP-10), CXCL11 (I-TAC) and IL-10 following the manufacturer’s instructions. Assay controls included kit standards and Multiplex controls.

Plasma concentrations of IL-18 (R&D systems, Minneapolis, MN, USA), IL-6 (Immunotools, Friesoythe, Germany) and TGFβ (Mabtech, Nacka Strand, Sweden) were determined using specific ELISA kits according to the manufacturers’ instructions and using the specific standard curves of recombinant molecules.

### 2.4. Statistical Analysis

Statistical analyses were performed using Graph Pad Prism 9 software (Graphpad, Boston, MA, USA). The Kolmogorov–Smirnov test was applied to test the normal distribution of the data. To describe our population, numbers and percentages were used for qualitative variables, and quantitative variables with non-normal distributions were reported as medians (interquartile range) (IQR). Student’s t or the Mann–Whitney tests were used for the comparison of variables between groups according to normal distribution. The comparisons of three or more groups were analysed with the non-one-way analysis of variance (ANOVA) Kruskal–Wallis test and the Dunn test. Pearson’s or Spearman’s coefficients were used for study cytokine correlations. Correlation matrices were drawn employing an R package called “corrplot”. Fisher and Chi-square tests were used for the comparison of frequencies between clusters of patients and the log-rank Mantel–Cox test was used to analyse differences between clusters in the staircase analysis. The area under the curve (AUC) of the receiver operating characteristic (ROC) curve was used to identify the most accurate predictive biomarkers of serious irAE development. A classification decision tree model was formulated to determine the optimal clustering variables in pre-irAE samples using the CHAID algorithm. All *p*-values lower than 0.05 were considered statistically significant. 

A network analysis to study the relationship between the analysed variables in a network as a whole was conducted with JASP 0.18.1 software (Amsterdam, The Netherlands). Networks are defined as a collection of interconnected components. The analysed chemokines and cytokines are represented by nodes, and the edges indicate the full conditional association between two nodes after conditioning on all of the other nodes in the network. The network structure underwent characterization through network centrality indices, specifically strength, closeness and betweenness [28,29].

We carried out a scalable cluster analysis as an exploratory technique to identify homogenous groups (clusters) of cases. Using the SPSS v.21 software (IBM, Chicago, IL, USA), a two-step clustering was performed to group cases based on the distribution of CXCL9, CXCL10, CXCL11, IL-18, IL-10, IL-6 and TGFβ concentrations in irAE samples using log-likelihood distance measures. The optimal number of clusters was automatically selected by an algorithm based on Akaike’s information Criterion. The resulting clusters distributed the patients in Cluster 1 (C1) n = 18, Cluster 2 (C2) n = 17 and Cluster 3 (C3) n = 6, according to 5 variables, CXCL9, CXCL10, CXCL11, IL-18 and IL10, while TGFβ and IL-6 variables were excluded by the algorithm. A dendrogram with the resulting distribution of cases was also represented. The five variables included produced a silhouette coefficient = 0.7 indicative of good data partitioning. 

## 3. Results

### 3.1. Patient Characteristics and Types of irAEs

Table 1 displays patient characteristics. The median age was 69 years, with a male predominance (70.8%). The distribution of tumour types was as follows: non-small cell lung cancer (NSCLC) was the most prevalent, affecting in ninety-one (69.7%) patients, followed by melanoma with twenty (14.1%) cases, renal cancer with ten (7.5%) cases, head and neck cancer with nine (6.7%) cases and bladder cancer with four (2.9%) cases. Regarding the treatment, 64 (47.7%) patients received PD-(L)1 inhibitors as a first-line treatment whereas 57 (42.5%) patients commenced it as a second-line or later stage treatment. In addition, five (3.7%) patients received PD-(L)1 inhibitors as an adjuvant therapy and eight (5.9%) as maintenance therapy following chemoradiation. PD-(L)1 inhibitors were administered as monotherapy in 104 (77.6%) patients, in combination with another immunotherapy agent in 19 (14.2%) patients and with chemotherapy in 11 (8.2%) patients.

As shown in Table 1, there were no significant differences in demographic, clinical and treatment characteristics between patients who developed serious irAEs, mild irAEs and no irAEs. 

Eighty-one patients (60.4%) developed irAEs. Sixty-five (48.5%) patients developed mild irAEs (grades 1–2) and 16 (11.9%) patients developed serious irAEs (grades 3–4) (Figure 1). No grade 5 irAEs occurred in this cohort. Patients with serious irAEs had received Nivolumab (n = 3), Pembrolizumab (n = 5), Atezolizumab (n = 2), Durvalumab (n = 1) and Nivolumab in combination with Ipilimumab (n = 3). Concerning the type of irAEs (Table 2), dermatological irAEs including rash and pruritus were the most frequent manifestations and were observed in 34 (26.1%) patients, followed by endocrine dysfunction in 14 (10.5%) patients and hepatitis in 12 (8.9%). The most severe irAEs (grades ≥ 3) affect hepatic, digestive, respiratory and endocrine systems. Table 2 shows the type, frequency and the moment of the appearance of irAEs developed in the different groups and in the total number of patients.

### 3.2. Changes in the Levels of Cytokines during ICI Treatment According to irAE Severity

**Figure 2:**
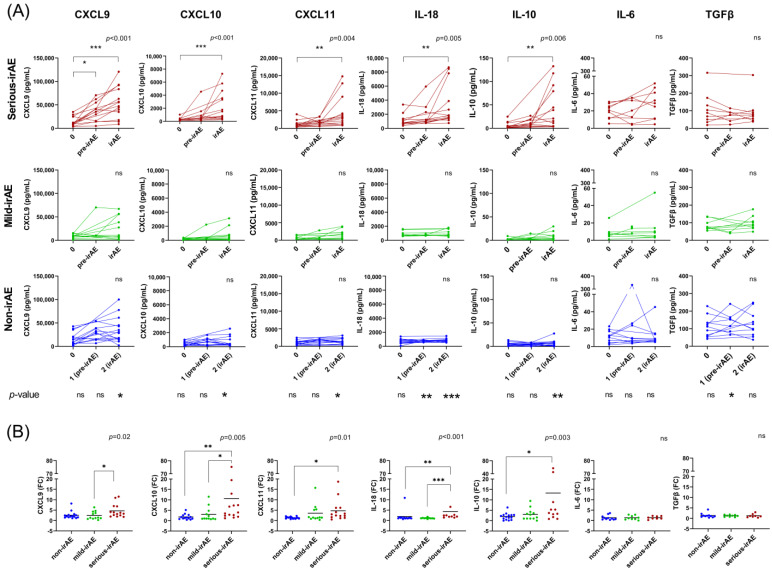
Changes in the levels of IFN-inducible chemokines and cytokines in serious, mild and non-irAE patients. (**A**) Monitoring of CXCL9, CXCL10, CXCL11, IL-18, IL-10, IL-6 and TGFβ levels at pre-treatment samples (0), samples before the irAE development (pre-irAE) and in the moment of irAE onset (irAE) in serious-irAE (red), mild-irAE (green) and non-irAE (blue) patients. Comparisons (*p*-values) between groups at different time points are shown. (**B**) Changes in IFN-inducible chemokines and cytokines at the onset of the irAE compared to baseline. Kruskal–Wallis and Dunn tests have been used for group comparisons. * *p* < 0.05; ** *p* < 0.01; *** *p* < 0.001; ns: non-significant.

In Figure 2, we show the levels of the IFN-inducible chemokines and related cytokines in the plasma of serious-irAE, mild-irAE and non-irAE patients. We did not observe any differences in pre-treatment sample levels of CXCL9, CXCL10, CXCL11, IL-18, IL-10, IL-6 nor TGFβ between groups. However, there were significant differences in CXCL9, CXCL10, CXCL11, IL-18 and IL-10 levels when comparing them across the three groups in the irAE samples (Figure 2A). We also observed significant differences in pre-irAE samples, specifically in the levels of IL-18 and TGFβ between the groups.

Patients in mild-irAE and non-irAE groups showed a non-significant increase in CXCL9, CXCL10 and IL-10 levels in irAE samples compared to pre-treatment samples, while the serious-irAE group exhibited a higher increase in the levels of CXCL9, CXCL10, CXCL11, IL-18 and IL-10 levels than other groups (Figure 2B). 

During the follow-up, sixteen patients received steroids following toxicity, while eight patients were administered steroids for disease management. We did not observe differences in cytokine levels between the samples of patients who received steroids before cytokine determination (n = 3) and those who did not.

### 3.3. Interplay of Cytokines in irAE Samples, According to the Severity of the irAE

**Figure 3:**
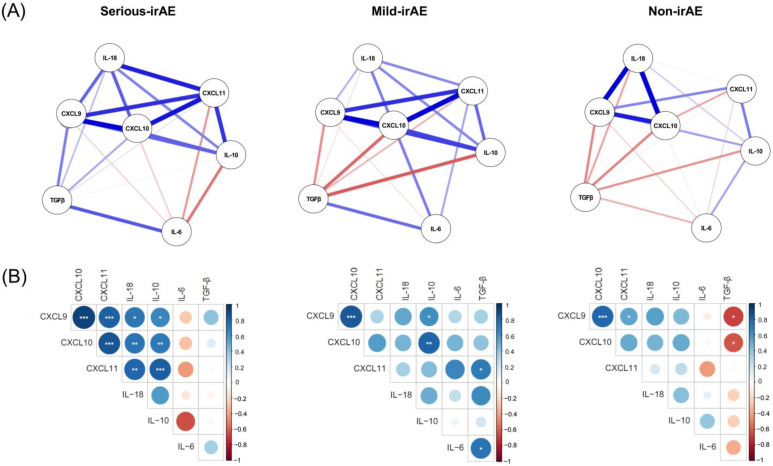
Relationship between IFN-inducible chemokines and cytokines at the moment of irAE onset. (**A**) Network analysis shows the proteins CXCL9, CXCL10, CXCL11, IL-18, IL-10, IL-6 and TGF-β represented by nodes, with the relationships among them depicted by edges, in serious-irAE, mild-irAE and non-irAE patients. The colour and thickness of the edges represent the direction and strength of the relationship and the centrality of a protein provides insight into the relative importance of this protein in the network. (**B**) Matrix correlation between CXCL9, CXCL10, CXCL11, IL-18, IL-10, IL-6 and TGF-β in the three groups of patients. The size and colour of the circles represent the correlation coefficient. Spearman correlations have been evaluated: * *p* < 0.05; ** *p* < 0.01; and *** *p* < 0.001.

We next conducted a network analysis to explore the relationship among the studied cytokines in irAE samples. Figure 3A shows the different networks and patterns observed in serious-irAE, mild-irAE and non-irAE groups. The central role of CXCL10 showed the relative importance of this protein in the network context. We observed a close and strong relationship involving CXCL9, CXCL10, CXCL11, IL-18 and IL-10 that conformed a node in the network in patients with irAEs. This node was not observed in patients without irAEs. Additionally, in cases of serious irAEs, unlike patients in mild-irAE and non-irAE groups, the pattern of TGFβ’s relationship with the rest of the studied proteins was different. The centrality indices in the network analysis (strength, closeness and betweenness) and the expected influence of the proteins in the three groups of patients are shown in Supplementary Figure S1. 

These results were in line with the correlation patterns shown in Figure 3B. In patients with serious irAEs, IFN-inducible chemokines CXCL9, CXCL10 and CXCL11 showed significant correlation among them, and also correlated with IL-18 and IL-10 in irAE samples (Figure 3B) but not in pre-treatment and pre-irAE samples. On the other hand, the non-irAE group showed a direct correlation of CXCL9 with CXCL10 and CXCL11 but none of the IFN-inducible chemokines significantly correlated with IL-10 and IL-18. Furthermore, inverse correlations between TGFβ and IFN-inducible chemokines were observed only in the non-irAE group, but not in the serious-irAE or mild-irAE groups. 

### 3.4. Clustering of irAE Samples Based on Cytokines and Their Association with Severity and Type of irAE

**Figure 4:**
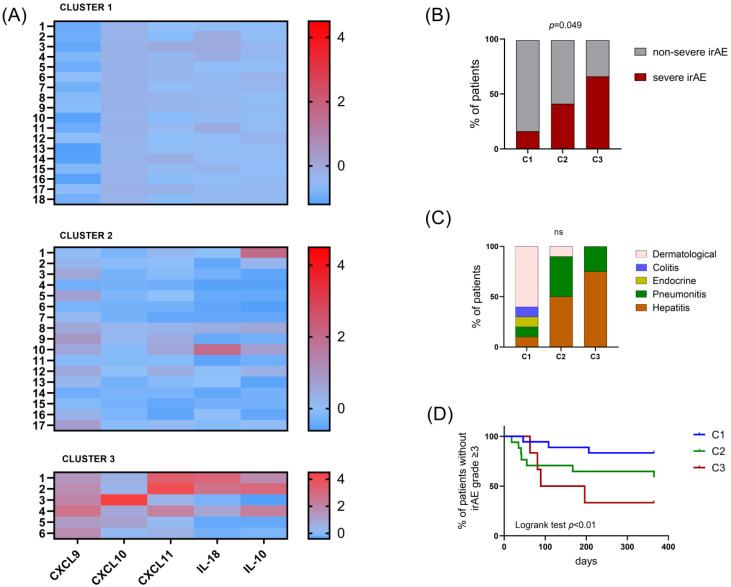
Comparison of cytokine and chemokine levels and type and severity of the irAE between clusters of patients. (**A**) Heat map shows normalized Z-scored values of CXCL9, CXCL10, CXCL11, IL-18 and IL-10 in each cluster of patients. Comparison of the three clusters of patients according to (**B**) severity of irAE and (**C**) type of irAE. (**D**) Staircase shows the probability of developing a serious irAE in the three clusters. Chi-square was used for the comparison of frequencies between clusters and the log-rank Mantel–Cox test was used for the staircase analysis.

Patients were distributed in Cluster 1 (C1) n = 18, Cluster 2 (C2) n = 17 and Cluster 3 (C3) n = 6 according to the profiles of cytokines and chemokines in irAE samples (Figure 4). Levels of the IFN-inducible chemokines IL-10 and IL-18 were significantly different between clusters (Supplementary Figure S2). The highest levels of CXCL9, CXCL10, CXCL11, IL-18 and IL-10 were observed in C3, while the lowest were observed in C1. There were no differences in TGFβ and IL-6 between clusters. Normalized Z-scored values in the three clusters are shown in Figure 4A. 

When we analysed the relationship between clusters and clinical outcomes (Figure 4B–D), we observed a higher proportion of patients who developed serious irAEs in C3 (66.7% in C3, 41.1% in C2 and 16.6% in C1, *p* = 0.04). In this cluster, there was also a higher proportion of patients experiencing more than one irAE, and later irAE onset. In addition, none of the patients with mild-grade dermatological or endocrine or colitis irAE were included in C3. Only hepatitis and pneumonitis irAEs comprised C3. 

Regarding the type of irAE, as shown in Supplementary Figure S3, the highest levels of IFN-inducible chemokines, IL-18 and IL-10, were observed in patients who developed hepatitis and pneumonitis. In severe hepatitis there was a trend towards higher levels of cytokines compared to mild hepatitis, although the sample size was not enough to reach statistical significance. Given that the most frequent tumour in our cohort was NSCLC, we additionally analysed cytokine levels separately in this kind of tumour. We observed similar levels of cytokines when comparing NSCLC to other tumours (Supplementary Figure S3). Non-significant differences were found in cytokine and chemokine levels among the most frequent treatments and between the most frequent tumour types (Supplementary Table S1). 

### 3.5. Pre-irAE Cytokine Levels as Predictive Markers for the Development of Serious irAEs

**Figure 5:**
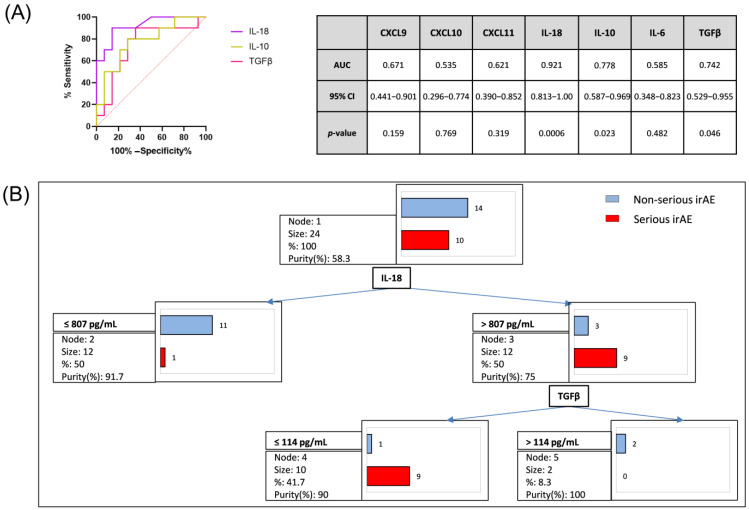
Predictive value of plasmatic levels of IFN-inducible chemokines and cytokines in pre-irAE samples. (**A**) ROC curve analysis shows sensitivity and specificity and area under the ROC curve (AUC) for IL-18, TGFβ, IL-10, CXCL9, CXCL10, CXCL11 and IL-6 as predictive biomarkers of serious irAE development. The parameters with the highest AUC values are represented in the graph. (**B**) Decision tree model where serious irAE patients (red) are classified as plasmatic levels of IL-18 > 807 pg/mL and TGFβ ≤ 114 pg/mL in 90% of cases or levels of IL-18 < 807 pg/mL and TGFβ < 50 pg/mL in 10% of cases. In contrast, non-serious-irAE patients (blue) were classified as IL-18 ≤ 807 pg/mL in 78.5% of cases.

To determine if pre-irAE cytokines could predict the development of serious irAEs, we performed an ROC analysis. Figure 5A shows sensitivity and specificity (AUC) for CXCL9, CXCL10, CXCL11 IL-18, TGFβ, IL-10 and IL-6 as predictive biomarkers of serious irAE development. IL-18 had the greatest ability to identify patients at risk for serious irAEs, with the highest area under ROC curve (AUC 0.921), followed by IL-10 (AUC 0.778) and TGFβ (AUC 0.742). 

We also applied a decision tree model to determine the best variables to discriminate between serious-irAE and mild-irAE/non-irAE patients in pre-irAE samples. The model showed that serious-irAE patients had plasmatic levels of IL-18 > 807 pg/mL and TGFβ < 114 pg/mL in pre-irAE samples in 90% of cases (nine patients). Only 10% of cases (one patient) had levels of IL-18 < 807 pg/mL. On the other hand, mild-irAE/non-irAE patients had IL-18 < 807 pg/mL in 78.5% of cases (Figure 5B). Additionally, the calculated TGFβ/IL-18 ratio exhibited significant differences among the non-irAE, mild-irAE and serious-irAE groups in irAE samples (0.12, 0.10 and 0.02, respectively; *p* < 0.01), as well as in pre-irAE samples (0.15, 0.16 and 0.05, respectively; *p* = 0.01).

## 4. Discussion

In this study, we observed significant differences in the levels of IFN-related cytokines between the serious, mild and non-irAE groups of patients, both in irAE and pre-irAE samples. Network and correlation analyses highlighted the central role of CXCL10 and distinctive patterns in serious irAE cases. The categorization of patients based on cytokine and chemokine profiles revealed higher proportions of serious irAEs in specific clusters. Importantly, IL-18 and TGFβ emerged as promising predictive biomarkers for serious irAE development. 

The absence of baseline differences in cytokine expression between groups suggests a lack of pre-existing predisposition to develop irAEs and their severity, although confirmation with larger cohorts is needed. There are controversial results from studies that examined cytokine expression at baseline based on irAE severity. Similar to our findings, Tyan et al. analysed a total of 34 cytokines, including CXCL10 and IL-6, and found no differences at baseline between patients developing grade 3–4 irAEs compared to grades 1–2 [30]. Another study [31], using a panel of 65 cytokines, revealed differences in 11 cytokines in serious irAEs, but the authors, similarly to us, did not find differences in CXCL10. On the other hand, some studies have identified lower baseline levels of CXCL9/10/11 [32] and IL-6 [33] or higher IL-10 [34] in patients with irAEs compared with those without irAEs. However, in these studies, data were not analysed according to irAE severity and heterogeneity in patient inclusion and ICI treatment was observed. These differences may explain the apparent divergence across different studies. 

When we analysed changes during the follow-up period, progressive increases in CXCL9, CXCL10, CXCL11 and IL-18 were observed leading up to the irAE onset, especially in samples from patients with serious irAEs. The upregulation of IFN-related chemokines after ICI treatment has been shown in other studies [15,30,32,35,36]. A possible mechanism behind our findings is that the interaction between CXCL9/10/11 and their receptor CXCR3 on activated T-cells, monocytes and neutrophils is involved in their trafficking and establishment of inflammation in peripheral sites. This suggests that not only T-cells, but also monocytes and neutrophils, could contribute to tissue damage through this mechanism [37,38,39,40]. CXCR3 is also upregulated in inflammatory conditions [41]. The higher increase in these IFN-inducible chemokines found in the serious irAE group may reflect a higher IFN-inducible inflammation in these patients. It is well established that IFN-γ signalling promotes the expression of PD-L1 in the tumour microenvironment [42,43,44,45], and blocking the PD-1/PD-L1 axis could potentially enhance IFN-mediated inflammatory responses [46]. On the other hand, not all patients with serious irAEs in our cohort exhibited this upregulation throughout the follow-up. The variation among patients may stem from the presence of additional inflammatory mechanisms not IFN-related, and/or other anti-inflammatory pathways involving immune-suppressor cells or mediators, where blocking the PD-1/PD-L1 axis alone may not be sufficient to counteract IFN-mediated inflammation. 

In addition to IFN-inducible chemokines, we have also analysed other related cytokines in this study. The network and correlation analyss revealed a strong relationship among CXCL9, CXCL10, CXCL11, IL-18 and IL-10 in patients at the onset of serious irAEs, and a loss of inverse correlation between IFN-related cytokines and TGFβ. In line with our findings, IL-10 upregulation after one cycle of ICI treatment has been positively associated with the occurrence of irAEs [34]. Studies have associated lower levels of TGFβ with higher IFNγ responses [47,48] and higher ICI efficacy [49,50]. All of these findings suggest that the inflammatory process that occurs during the irAE impairs the capacity for regulatory control processes. 

Concerning IL-18, the direct correlation with IFN-inducible chemokines observed in the serious irAE group was to be expected, given that the primary role of IL-18 is to stimulate the secretion of IFNγ from Th1 cells [51,52]. However, Wang et al. [17] demonstrated an indirect correlation between CXCL10 and IL-18 changes after ICI treatment, but, differently from us, they analysed a whole cohort of NSCLC patients treated with ICI regardless the presence and/or severity of irAEs. In fact, they did not observe differences in IL-18 levels during ICI treatment between patients with and without irAEs. Our analysis goes further, distinguishing between the severities of the irAEs and observing the elevation of both IL-18 and IFN-inducible chemokines in the serious forms only, which reinforces the hypothesis that these cytokines play a crucial role in the severity of these immune dysregulation events. 

Moreover, analysing the pre-irAE samples through ROC analysis and decision tree modelling allowed us to identify high IL-18 and low TGFβ levels as promising biomarkers to predict serious irAEs. We hypothesized that the dysregulation driving irAE development can be produced as a consequence of an early IL-18 upregulation. Other studies have identified IL-18 as an inducer of Th-1 autoimmune diseases [53]. IL-18 could enhance IFNγ responses, and subsequently CXCL9/10/11 expression, which promotes the migration of CXCR3+ cells to different tissues. In addition, low levels of TGFβ could contribute to an increase in CXCR3 expression, as shown in TGFβ receptor I-deficient T cells [54]. Subsequently, localized inflammatory amplification loops can be generated within target organs, contributing to tissue damage and serious irAE development. The mechanisms underlying early IL-18 and TGFβ dysregulation need to be further investigated.

We observed that patients who received steroids or chemotherapy agents in combination with ICIs, exhibited comparable cytokine levels to those who received ICIs alone. Chemotherapy treatment may enhance the efficacy of ICIs by activating IFN signalling, as evidenced by the elevation of type I IFN observed in certain chemotherapy treatments [55]. However, the low number of patients who received chemotherapy or steroids in our cohort did not allow us to determine whether these agents have influenced our results. 

Since the onset of the irAEs is unpredictable and can appear at any moment during or after the course of the treatment, the precise selection of samples at the time of irAE onset, as well as before irAE in each patient individually, is a notable strength of this study, as the results obtained more accurately show the ongoing biological processes within each individual. Furthermore, patients included are not limited to a single type of tumour, which allows us to associate these results with irAEs independently of this parameter.

Our study presents some limitations. Despite studying a cohort of over 130 patients, since the frequency of serious irAEs is low, the small sample size of the studied groups did not permit us to analyse whether the patient intrinsic characteristics, agents used, type of tumour, type of irAE and related parameters may be influencing our findings. We are planning to validate our results with a larger patient cohort, thereby strengthening our conclusions. Another limitation is that we have not investigated whether the observed results persist over time. Further studies analysing IFN-related cytokines and chemokines after the discontinuation of PD-(L)1 inhibitors or steroid treatments would be of great interest.

## 5. Conclusions

In conclusion, irAEs are complex immune-mediated processes in which different proteins with distinct kinetics could participate in “functional networks”. Monitoring these proteins throughout the treatment, not individually, but considering them as a whole, could anticipate the occurrence of serious irAEs, facilitating their prediction and therefore improving the management of ICI treatments by enabling more timely and targeted interventions in the case of adverse events. Our findings offer valuable insights into the intricate immune responses associated with irAEs and propose potential predictive markers for their severity.

## Figures and Tables

**Table 1:** Characteristics of patients and comparison among patients with serious, mild and no irAEs.

	All Patients, n = 134	Serious irAE n = 14	Mild irAE n = 12	No irAE n = 15	*p*-Value
**Sex male, n (%)**	95 (70.8)	10 (71.4)	9 (75)	12 (80)	0.86
**Age, median (range)**	69 (37–89)	68.5 (45–83)	67 (60–70)	67 (43–86)	0.94
**Smoker, n (%)**	75 (55.9)	11 (78.5)	7 (58.3)	12 (80)	0.38
**PD-L1 expression, n (%) †**					
Negative (<1%)	12 (18.8)	2 (20)	0 (0)	1 (10)	0.59
Low (1–49%)	23 (35.9)	5 (50)	1 (25)	4 (40)	
High (>50%)	29 (45.3)	3 (30)	3 (75)	5 (50)	
**Tumour type, n (%)**					
NSCLC	91 (67.9)	9 (64.3)	8 (66.7)	10 (66.7)	0.52
Melanoma	20 (14.9)	1 (7.1)	3 (25)	2 (13.3)	
Renal	10 (7.5)	3 (21.4)	0 (0)	2 (13.3)	
Head and neck	9 (6.7)	0 (0)	1 (8.3)	0 (0)	
Urothelial	4 (2.9)	1 (7.1)	0 (0)	1 (6.7)	
**Line of treatment, n (%)**					
1st line	64 (47.8)	7(50)	5 (41.7)	8(53.3)	0.96
≥2nd line	57 (42.5)	5 (35.7)	5 (41.7)	6 (40)	
Adjuvant	5 (3.7)	1 (7.1)	1 (8.3)	1 (6.7)	
Maintenance	8 (5.9)	1 (7.1)	1 (8.3)	0 (0)	
**ICI agent, n (%)**					
Anti-PD-1	91 (67.9)	11 (78.5)	9 (75)	10 (66.7)	0.75
Anti-PD-L1	43 (32.1)	3 (21.4)	3 (25)	5 (33.3)	
**ICI schedule, n (%)**					
Monotherapy	104 (77.6)	9 (64.3)	11 (91.7)	13 (86.7)	0.23
Combination with immunotherapy	19 (14.2)	4 (28.6) *	0 (0)	2 (13.3) **	
Combination with chemotherapy	11 (8.2)	1 (7.1)	1 (8.3)	0 (0)	

Chi-square was used for frequencies comparison between serious, mild and non-irAE groups. † PD-L1 expression available only in 64 patients (10, 4 and 10 patients in each group, respectively). Percentages are in reference to available results. * Oclelimumab (n = 1) and Ipilimumab (n = 3). ** Eftilagimod.

**Table 2:** Description and frequency of irAEs.

	Number of Patients	Onset Time, Median Days (Range)	Number of Patients	Onset Time, Median Days (Range)	Number of Patients	Onset Time, Median Days (Range)
**Type of irAEs**	**All Grades (n = 81)**		**Serious Grades (n = 14)**		**Mild Grades (n = 12)**	
**Dermatological**						
Rash	11	14 (1–98)	0	_	2	25 (9–41)
Pruritus	24	42 (2–334)	0	_	3	42 (21–69)
**Colitis**	3	108 (18–297)	1	108	0	
**Endocrine**						_
Hypothyroidism	9	46 (20–86)	1	46	0	_
Hyperthyroidism	3	60 (42–206)	1	206	0	_
Hypophysitis	1	80	0	_	0	_
Diabetes mellitus	1	41	0	_	0	_
**Pneumonitis**	6	83 (55–159)	2	68 (55–81)	4	109 (62–159)
**Hepatitis**	12	52 (6–364)	9	63 (18–364)	3	14 (6–201)
**Arthralgia**	4	31 (1–140)	0	_	0	_
**Other**						
Mucositis	2	158 (11–306)	0	_	0	_
Xerosis	2	14 (1–27)	0	_	0	_
Vitiligo	1	217	0	_	0	_
Psoriasis	1	11	0	_	0	_
Neuropathy	1	45	0	_	0	_

Serious irAEs include any grade ≥ 3 irAE and mild irAEs include grade ≤ 2 irAEs, according to CTCAE 5.0.

## Data Availability

The experimental data generated during the current study are available from the corresponding author on reasonable request.

## Supplementary Materials

The following supporting information can be downloaded at: https://www.mdpi.com/2072-6694/16/9/1759/s1, Figure S1: Centrality indices for the estimated networks. Centrality indices of CXCL9, CXCL10, CXCL11, IL18, IL-10, IL-6 and TGFβ proteins in serious-irAE, mild-irAE and non-irAE patients shown in Figure 3A. Figure S2: Cluster analysis according to IFN-inducible chemokines and cytokines at irAE onset. (A) Dendrogram shows the classification of irAE and non-irAE patients in 3 clusters, according to (B) levels of plasmatic soluble factors CXCL9, CXCL10 (IP-10), CXCL11, IL-18, IL-10, IL-6 and TGF-β. Kruskal-Wallis test was used for comparison between clusters. ** *p* < 0.01; *** *p* < 0.001; **** *p* < 0.0001. Figure S3: Levels of CXCL9, CXCL10, CXCL11, IL-18, IL-10, IL6 and TGFβ at the onset of the irAE according to type and severity. Patients with NSCLC (most frequent tumour type) are specified.

## References

[1] Nixon N.A., Blais N., Ernst S., Kollmannsberger C., Bebb G., Butler M., Smylie M., Verma S. (2018). Current landscape of immunotherapy in the treatment of solid tumours, with future opportunities and challenges. Curr. Oncol..

[2] Postow M.A., Sidlow R., Hellmann M.D. (2018). Immune-Related Adverse Events Associated with Immune Checkpoint Blockade. N. Engl. J. Med..

[3] Naidoo J., Murphy C., Atkins M.B., Brahmer J.R., Champiat S., Feltquate D., Krug L.M., Moslehi J., Pietanza M.C., Riemer J. (2023). Society for Immunotherapy of Cancer (SITC) consensus definitions for immune checkpoint inhibitor-associated immune-related adverse events (irAEs) terminology. J. Immunother. Cancer.

[4] von Itzstein M.S., Khan S., Gerber D.E. (2020). Investigational Biomarkers for Checkpoint Inhibitor Immune-Related Adverse Event Prediction and Diagnosis. Clin. Chem..

[5] Ayers M., Lunceford J., Nebozhyn M., Murphy E., Loboda A., Kaufman D.R., Albright A., Cheng J.D., Kang S.P., Shankaran V. (2017). IFN-γ-related mRNA profile predicts clinical response to PD-1 blockade. J. Clin. Investig..

[6] Martínez-Sabadell A., Arenas E.J., Arribas J. (2022). IFNγ Signaling in Natural and Therapy-Induced Antitumor Responses. Clin. Cancer Res..

[7] Baechler E.C., Batliwalla F.M., Karypis G., Gaffney P.M., Ortmann W.A., Espe K.J., Shark K.B., Grande W.J., Hughes K.M., Kapur V. (2003). Interferon-inducible gene expression signature in peripheral blood cells of patients with severe lupus. Proc. Natl. Acad. Sci. USA.

[8] Assassi S., Mayes M.D., Arnett F.C., Gourh P., Agarwal S.K., McNearney T.A., Chaussabel D., Oommen N., Fischbach M., Shah K.R. (2010). Systemic Sclerosis and Lupus: Points in an Interferon-Mediated Continuum Shervin. Arthritis Rheum..

[9] Kimoto O., Sawada J., Shimoyama K., Suzuki D., Nakamura S., Hayashi H., Ogawa N. (2011). Activation of the interferon pathway in peripheral blood of patients with Sjögren’s syndrome. J. Rheumatol..

[10] Perez R.K., Gordon M.G., Subramaniam M., Kim M.C., Hartoularos G.C., Targ S., Sun Y., Ogorodnikov A., Bueno R., Lu A. (2022). Single-cell RNA-seq reveals cell type-specific molecular and genetic associations to lupus. Science.

[11] Kim H., De Jesus A.A., Brooks S.R., Liu Y., Huang Y., Vantries R., Montealegre Sanchez G.A., Rotman Y., Gadina M., Goldbach-Mansky R. (2018). Development of a Validated Interferon Score Using NanoString Technology. J. Interf. Cytokine Res..

[12] Rice G.I., Melki I., Frémond M.L., Briggs T.A., Rodero M.P., Kitabayashi N., Oojageer A., Bader-Meunier B., Belot A., Bodemer C. (2016). Assessment of Type I Interferon Signaling in Pediatric Inflammatory Disease. J. Clin. Immunol..

[13] Groom J. (2008). CXCR3 in T cell function. Bone.

[14] Tokunaga R., Zhang W., Naseem M., Puccini A., Berger M.D., Soni S., McSkane M., Baba H., Lenz H.J. (2018). Target for Novel Cancer Therapy. Cancer Treat. Rev..

[15] Wang H., Li S., Wang Q., Jin Z., Shao W., Gao Y., Li L., Lin K., Zhu L., Wang H. (2021). Tumor immunological phenotype signature-based high-throughput screening for the discovery of combination immunotherapy compounds. Sci. Adv..

[16] Chow M.T., Ozga A.J., Servis R.L., Frederick D.T., Jennifer A., Fisher D.E., Freeman G.J., Boland G.M., Andrew D. (2020). Intratumoral activity of the CXCR3 chemokine system is required for the efficacy of anti- PD-1 therapy. Immunity.

[17] Wang Y., Chen H., Zhang T., Yang X., Zhong J., Wang Y., Chi Y., Wu M., An T., Li J. (2021). Plasma cytokines interleukin-18 and C-X-C motif chemokine ligand 10 are indicative of the anti-programmed cell death protein-1 treatment response in lung cancer patients. Ann. Transl. Med..

[18] Haratani K., Hayashi H., Chiba Y., Kudo K., Yonesaka K., Kato R., Kaneda H., Hasegawa Y., Tanaka K., Takeda M. (2018). Association of immune-related adverse events with nivolumab efficacy in non-small cell lung cancer. JAMA Oncol..

[19] Indini A., Di Guardo L., Cimminiello C., Prisciandaro M., Randon G., De Braud F., Del Vecchio M. (2019). Immune-related adverse events correlate with improved survival in patients undergoing anti-PD1 immunotherapy for metastatic melanoma. J. Cancer Res. Clin. Oncol..

[20] Riudavets M., Mosquera J., Garcia-Campelo R., Serra J., Anguera G., Gallardo P., Sullivan I., Barba A., del Carpio L., Barnadas A. (2020). Immune-Related Adverse Events and Corticosteroid Use for Cancer-Related Symptoms Are Associated with Efficacy in Patients with Non-small Cell Lung Cancer Receiving Anti-PD-(L)1 Blockade Agents. Front. Oncol..

[21] Hu W., Wang G., Wang Y., Riese M.J., You M. (2020). Uncoupling Therapeutic Efficacy from Immune-Related Adverse Events in Immune Checkpoint Blockade. iScience.

[22] Zhou X., Yao Z., Yang H., Liang N., Zhang X., Zhang F. (2020). Are immune-related adverse events associated with the efficacy of immune checkpoint inhibitors in patients with cancer? A systematic review and meta-analysis. BMC Med..

[23] Landy E., Carol H., Ring A., Canna S. (2024). Biological and clinical roles of IL-18 in inflammatory diseases. Nat. Rev. Rheumatol..

[24] Sanjabi S., Oh S.A., Li M.O. (2017). Regulation of the immune response by TGF-β: From conception to autoimmunity and infection. Cold Spring Harb. Perspect. Biol..

[25] Wu Y.R., Hsing C.H., Chiu C.J., Huang H.Y., Hsu Y.H. (2022). Roles of IL-1 and IL-10 family cytokines in the progression of systemic lupus erythematosus: Friends or foes?. IUBMB Life.

[26] Iyer S.S., Cheng G. (2012). Role of interleukin 10 transcriptional regulation in inflammation and autoimmune disease. Crit. Rev. Immunol..

[27] National Cancer Institute (US) (2017). Common Terminology Criteria for Adverse Events (CTCAE).

[28] Papachristou N., Barnaghi P., Cooper B., Kober K.M., Maguire R., Paul S.M., Hammer M., Wright F., Armes J., Furlong E.P. (2019). Network Analysis of the Multidimensional Symptom Experience of Oncology. Sci. Rep..

[29] Wang Y., Hu Z., Feng Y., Wilson A., Chen R. (2020). Changes in network centrality of psychopathology symptoms between the COVID-19 outbreak and after peak. Mol. Psychiatry.

[30] Tyan K., Baginska J., Brainard M., Giobbie-Hurder A., Severgnini M., Manos M., Haq R., Buchbinder E.I., Ott P.A., Hodi F.S. (2021). Cytokine changes during immune-related adverse events and corticosteroid treatment in melanoma patients receiving immune checkpoint inhibitors. Cancer Immunol. Immunother..

[31] Lim S.Y., Lee J.H., Gide T.N., Menzies A.M., Guminski A., Carlino M.S., Breen E.J., Yang J.Y.H., Ghazanfar S., Kefford R.F. (2019). Circulating cytokines predict immune-related toxicity in melanoma patients receiving anti-PD-1–based immunotherapy. Clin. Cancer Res..

[32] Khan S., Khan S.A., Luo X., Fattah F.J., Saltarski J., Gloria-McCutchen Y., Lu R., Xie Y., Li Q., Wakeland E. (2019). Immune dysregulation in cancer patients developing immune-related adverse events. Br. J. Cancer.

[33] Valpione S., Pasquali S., Campana L.G., Piccin L., Mocellin S., Pigozzo J., Chiarion-Sileni V. (2018). Sex and interleukin-6 are prognostic factors for autoimmune toxicity following treatment with anti-CTLA4 blockade. J. Transl. Med..

[34] Wang H., Zhou F., Zhao C., Cheng L., Zhou C., Qiao M., Li X., Chen X. (2022). Interleukin-10 Is a Promising Marker for Immune-Related Adverse Events in Patients With Non-Small Cell Lung Cancer Receiving Immunotherapy. Front. Immunol..

[35] Nuñez N.G., Berner F., Friebel E., Unger S., Wyss N., Gomez J.M., Purde M.T., Niederer R., Porsch M., Lichtensteiger C. (2023). Immune signatures predict development of autoimmune toxicity in patients with cancer treated with immune checkpoint inhibitors. Med.

[36] Peng W., Liu C., Xu C., Lou Y., Chen J., Yang Y., Yagita H., Overwijk W.W., Lizée G., Radvanyi L. (2012). PD-1 blockade enhances T cell migration to tumors by elevating IFN-γ inducible chemokines. Cancer Res..

[37] Ashoori M.D., Suzuki K., Tokumaru Y., Ikuta N., Tajima M., Honjo T., Ohta A. (2021). Inactivation of the PD-1-Dependent Immunoregulation in Mice Exacerbates Contact Hypersensitivity Resembling Immune-Related Adverse Events. Front. Immunol..

[38] Kim S.T., Chu Y., Misoi M., Suarez-Almazor M.E., Tayar J.H., Lu H., Buni M., Kramer J., Rodriguez E., Hussain Z. (2022). Distinct molecular and immune hallmarks of inflammatory arthritis induced by immune checkpoint inhibitors for cancer therapy. Nat. Commun..

[39] Hailemichael Y., Johnson D.H., Abdel-Wahab N., Foo W.C., Eddine-Bentebibel S., Daher M., Haymaker C., Wani K., Saberian C. (2022). Interleukin-6 blockade abrogates immunotherapy toxicity and promotes tumor immunity. Cancer Cell.

[40] Hirani D.V., Thielen F., Mansouri S., Danopoulos S., Vohlen C., Haznedar-Karakaya P., Mohr J., Wilke R., Selle J., Grosch T. (2023). CXCL10 deficiency limits macrophage infiltration, preserves lung matrix, and enables lung growth in bronchopulmonary dysplasia. Inflamm. Regen..

[41] Satarkar D., Patra C. (2022). Evolution, Expression and Functional Analysis of CXCR3 in Neuronal and Cardiovascular Diseases: A Narrative Review. Front. Cell Dev. Biol..

[42] Cha J.H., Chan L.C., Li C.W., Hsu J.L., Hung M.C. (2019). Mechanisms Controlling PD-L1 Expression in Cancer. Mol. Cell.

[43] Colli M.L., Hill J.L.E., Marroquí L., Chaffey J., Santos R.S.D., Leete P., Coomans A., Paula F.M.M., de Beeck A.O., Castela A. (2018). PDL1 is expressed in the islets of people with type 1 diabetes and is up-regulated by interferons-α and-γ via IRF1 induction. EbioMedicine.

[44] Garcia-diaz A., Sanghoon D., Moreno B.H., Saco J., Escuin-Ordinas H., Rodriguez G.A., Zaretsky J.M., Sun L., Hugo W., Wang X. (2017). Interferon Receptor Signaling Pathways Regulating PD-L1 and PD-L2 Expression. Cell Rep..

[45] Abiko K., Matsumura N., Hamanishi J., Horikawa N., Murakami R., Yamaguchi K., Yoshioka Y., Baba T., Konishi I., Mandai M. (2015). IFN-γ from lymphocytes induces PD-L1 expression and promotes progression of ovarian cancer. Br. J. Cancer.

[46] Gato-Cañas M., Zuazo M., Arasanz H., Ibañez-Vea M., Lorenzo L., Fernandez-Hinojal G., Vera R., Smerdou C., Martisova E., Arozarena I. (2017). PDL1 Signals through Conserved Sequence Motifs to Overcome Interferon-Mediated Cytotoxicity. Cell Rep..

[47] Yang S.C., Batra R.K., Hillinger S., Reckamp K.L., Strieter R.M., Dubinett S.M., Sharma S. (2006). Intrapulmonary administration of CCL21 gene-modified dendritic cells reduces tumor burden in spontaneous murine bronchoalveolar cell carcinoma. Cancer Res..

[48] Meadows S.K., Eriksson M., Barber A., Sentman C.L. (2006). Human NK cell IFN-γ production is regulated by endogenous TGF-β. Int. Immunopharmacol..

[49] Feun L.G., Li Y., Wu C., Wangpaichitr M., Jones P.D., Richman S.P., Madrazo B., Garcia-buitrago M., Martin P., Hosein P.J. (2020). Phase 2 Study of Pembrolizumab and Circulating Biomarkers to Predict Anticancer Response in Advanced, Unresectable Hepatocellular Carcinoma. Cancer.

[50] Mariathasan S., Turley S.J., Nickles D., Castiglioni A., Yuen K., Wang Y., Kadel Iii E.E., Koeppen H., Astarita J.L., Cubas R. (2018). TGF-β attenuates tumour response to PD-L1 blockade by contributing to exclusion of T cells. Nature.

[51] Esmailbeig M., Ghaderi A. (2017). Interleukin-18: A regulator of cancer and autoimmune diseases. Eur. Cytokine Netw..

[52] Timperi E., Focaccetti C., Gallerano D., Panetta M., Spada S., Gallo E., Visca P., Venuta F., Diso D., Prelaj A. (2017). IL-18 receptor marks functional CD8^+^ T cells in non-small cell lung cancer. Oncoimmunology.

[53] Oikawa Y., Shimada A., Kasuga A., Morimoto J., Osaki T., Tahara H., Miyazaki T., Tashiro F., Yamato E., Miyazaki J.I. (2003). Systemic Administration of IL-18 Promotes Diabetes Development in Young Nonobese Diabetic Mice. J. Immunol..

[54] Gunderson A.J., Yamazaki T., McCarty K., Fox N., Phillips M., Alice A., Blair T., Whiteford M., O’Brien D., Ahmad R. (2020). TGFβ suppresses CD8^+^ T cell expression of CXCR3 and tumor trafficking. Nat. Commun..

[55] Galluzzi L., Humeau J., Buqué A., Zitvogel L., Kroemer G. (2020). Immunostimulation with chemotherapy in the era of immune checkpoint inhibitors. Nat. Rev. Clin. Oncol..

