# Supplementary material for: Kinetics of IFNγ-Induced Cytokines and Development of Immune-Related Adverse Events in Patients Receiving PD-(L)1 Inhibitors
Source: Cancers (Basel). 2024 May 1;16(9):1759. doi: 10.3390/cancers16091759 (PMC11083441; doi:10.3390/cancers16091759)
Supplement: Supplementary file 1 [file cancers-16-01759-s001.zip › cancers-2918816-supplementary.pdf]

SUPPLEMENTARY MATERIALS

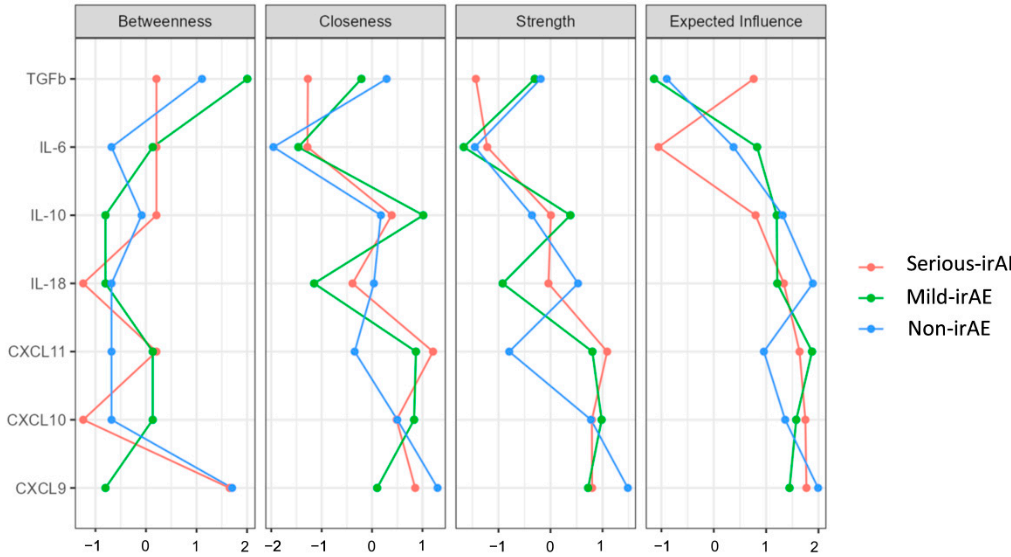

Figure supplementary S1: Centrality indices for the estimated networks. Centrality indices of CXCL9, CXCL10, CXCL11, IL18, IL-10, IL-6 and TGFβ proteins in serious-irAE, mild-irAE and non-irAE patients shown in Figure 3A.

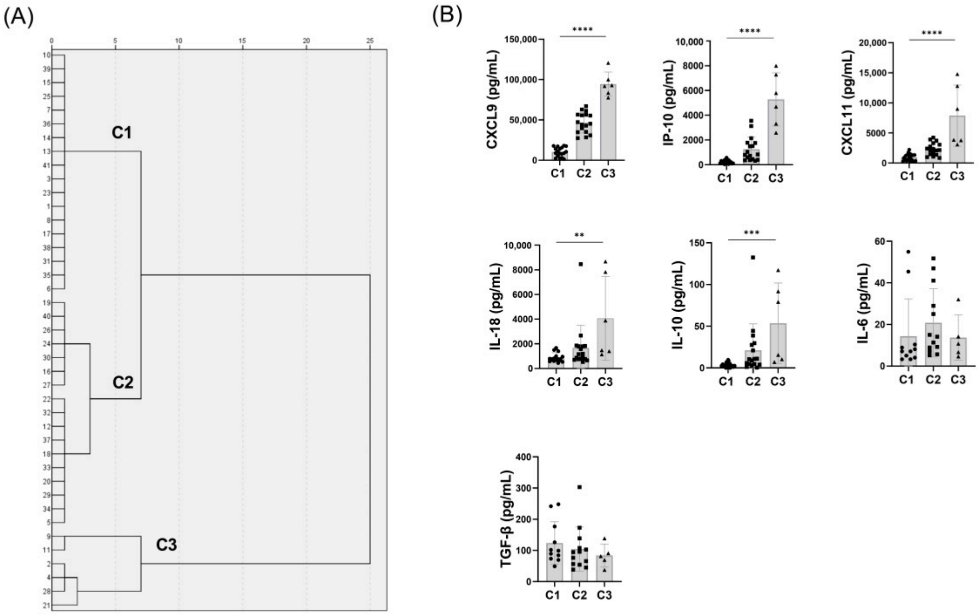

Figure supplementary S2: Cluster analysis according to IFN-inducible chemokines and cytokines at irAE onset. A) Dendrogram shows the classification of irAE and non-irAE patients in 3 clusters, according to B) levels of plasmatic soluble factors CXCL9, CXCL10 (IP-10), CXCL11, IL-18, IL-10, IL-6 and TGFβ. Kruskal-Wallis test was used for comparison between clusters. $^{**}p<0.01$; $^{***}p<0.001$; $^{****}p<0.0001$.

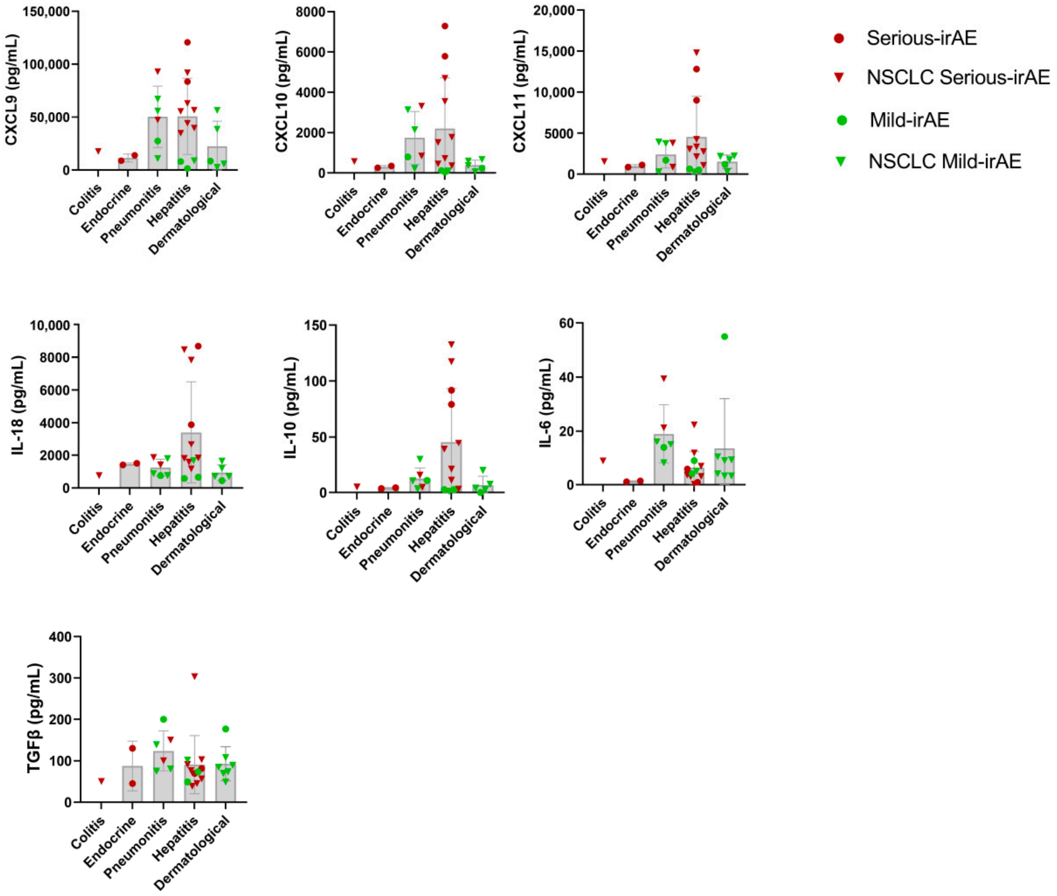

Figure supplementary S3: Levels of CXCL9, CXCL10, CXCL11, IL-18, IL-10, IL6 and TGFβ at the onset of the irAE according to type and severity. Patients with NSCLC (most frequent tumour type) are specified.

	CXCL9	CXCL10	CXCL11	IL-18	IL-10	IL-6	TGFβ
**Levels at baseline, according to tumour type, median (IQR) pg/mL**							
**NSCLC (n=27)**	10553<br>(8147-20,808)	267.5<br>(174.7-362)	920<br>(630.1-1491)	790.5<br>(578.6-1046)	2.9<br>(2.5-5.3)	12.7<br>(4.9-23.7)	94.8<br>(56.6-133.8)
**Melanoma (n=6)**	7947<br>(5461-10,767)	251.5<br>(126.7-383)	508.7<br>(294.9-1241)	678.7<br>(477.3-1823)	1.82<br>(1.14-4.63)	10.3<br>(7.3-11.7)	74<br>(58.13-96.8)
**Renal (n=5)**	1055<br>(6251-21,770)	337.5<br>(103.4-739)	709.2<br>(374.4-1383)	591.2<br>(480.9-1369)	1.61<br>(1.2-8.1)	14.4<br>(5.3-23.5)	78.3<br>(68.2-88.4)
**Urothelial (n=2)**	9982<br>(4438-15,526)	219.2<br>(204.2-233.6)	475.8<br>(450.8-500.7)	609.3<br>(480.9-737.7)	3.6<br>(1.2-6.0)	7.9<br>(4.6-11.4)	87.1<br>(75.2-99.1)
**Head and Neck (n=1)**	1322	48.8	410.7	824.2	2.5	8.4	135.2
**Levels at irAE onset, according to tumour type , median (IQR) pg/mL**							
**NSCLC (n=27)**	39,646<br>(17,352-61,366)	573.1<br>(366,4-1778)	1779<br>(1081-3084)	1144<br>(764.9-1670)	7.1<br>(3.0-15.6)	8.5<br>(5.5-26.0)	100.4<br>(66.2-147.4)
**Melanoma (n=6)**	17,959<br>(6661-54,840)	517.4<br>(125,2-2771)	1459<br>(466.3-4958)	700.5<br>(512.1-2771)	6.7<br>(1.8-40.5)	12.7<br>(9.2-27.8)	73.6<br>(53.3-87.5)
**Renal (n=5)**	35188<br>(11,467-88,614)	1492<br>(216-5419)	2120<br>(855.9-6161)	1500<br>(829.9-3281)	8.9<br>(1.8-65.2)	31.2<br>(10.7-51.7)	72.9<br>(69.5-76.4)
**Urothelial (n=2)**	13997<br>(1493-18,242)	213.7<br>(45.8-350.7)	639.3<br>(424.5-1121)	600<br>(578.9-1406)	4.2<br>(2.5-8.5)	6,9<br>(1.1-12.8)	81.5<br>(73.5-89.6)
**Head and Neck (n=1)**	1493	45.8	639.3	578.9	2.5	4.1	73.5
**Levels at irAE onset, according to treatment median (IQR) pg/mL**							
**Anti-PD1 (n=23)**	35,027<br>(8697-55,702)	407.2<br>(211.5-1578)	1586<br>(858.3-3067)	923.7<br>(684.5-1425)	3.7<br>(2.5-12.2)	9.1<br>(6.7-14.8)	90.4<br>(58.8-121.6)
**Anti-PDL1 (n=10)**	23316<br>(15,847-57565)	478.9<br>(355.8-1870)	1466<br>(633.2-3009)	794.4<br>(680.8-1593)	7.9<br>(6.5-10.1)	14.2<br>(7.0-45.4)	99.1<br>(69.5-241.7)
**Anti-PD(L)1 in combination with immunotherapy (n=6)**	54,368<br>(26,683-92,799)	945<br>(342.9-6167)	1950<br>(1057-9954)	1634<br>(830.5-5086)	5.1<br>(3.3-82.3)	8.2<br>(5.2-26.7)	75.4<br>(58.1-150.0)
**Anti-PD(L)1 in combination with chemotherapy (n=2)**	32,689<br>(8830-56,548)	1823<br>(94.3-6167)	1789<br>(255.5-3322)	2172<br>(1670-2674)	19.9<br>(1.1-38.7)	28.4<br>(5.0-51.8)	89.1<br>(76.5-101.7)

Table S1: Levels of IFN-related chemokines and cytokines according to tumour type and treatment. Levels of CXCL9, CXCL10, CXCL11, IL-18, IL-10, IL6 and TGFβ at baseline and at the onset of irAE according to tumour type, and at the onset of irAE according to treatment received. Median (IQR) values are shown. Kruskal-Wallis test was used for comparison between groups with sample sizes equal to or greater than n=5. Non-significant results were obtained.
